# The evaluation of pituitary damage associated with cardiac arrest: An experimental rodent model

**DOI:** 10.1038/s41598-020-79780-3

**Published:** 2021-01-12

**Authors:** Yu Okuma, Tomoaki Aoki, Santiago J. Miyara, Kei Hayashida, Mitsuaki Nishikimi, Ryosuke Takegawa, Tai Yin, Junhwan Kim, Lance B. Becker, Koichiro Shinozaki

**Affiliations:** 1 The Feinstein Institutes for Medical Research, Northwell Health, 350 Community Dr., Manhasset, NY 11030 USA; 2 Elmezzi Graduate School of Molecular Medicine at Northwell Health, Manhasset, NY USA; 3 Department of Emergency Medicine, Donald and Barbara Zucker School of Medicine at Hofstra/Northwell, Hempstead, NY USA

**Keywords:** Cell death in the nervous system, Pituitary gland, Translational research

## Abstract

The pituitary gland plays an important endocrinal role, however its damage after cardiac arrest (CA) has not been well elucidated. The aim of this study was to determine a pituitary gland damage induced by CA. Rats were subjected to 10-min asphyxia and cardiopulmonary resuscitation (CPR). Immunohistochemistry and ELISA assays were used to evaluate the pituitary damage and endocrine function. Samples were collected at pre-CA, and 30 and 120 min after cardio pulmonary resuscitation. Triphenyltetrazolium chloride (TTC) staining demonstrated the expansion of the pituitary damage over time. There was phenotypic validity between the pars distalis and nervosa. Both CT-proAVP (pars nervosa hormone) and GH/IGF-1 (pars distalis hormone) decreased over time, and a different expression pattern corresponding to the damaged areas was noted (CT-proAVP, 30.2 ± 6.2, 31.5 ± 5.9, and 16.3 ± 7.6 pg/mg protein, *p* < 0.01; GH/IGF-1, 2.63 ± 0.61, 0.62 ± 0.36, and 2.01 ± 0.41 ng/mg protein, *p* < 0.01 respectively). Similarly, the expression pattern between these hormones in the end-organ systems showed phenotypic validity. Plasma CT-proAVP (r = 0.771, *p* = 0.025) and IGF-1 (r = −0.775, *p* = 0.024) demonstrated a strong correlation with TTC staining area. Our data suggested that CA induces pathological and functional damage to the pituitary gland.

## Introduction

New insights into the pathophysiology of the brain after cardiac arrest (CA) are essential to improve mortality and morbidity of post-CA patients. A systemic ischemia–reperfusion injury triggers multiple organ-specific responses that process organ damage^1,2^. The pituitary gland, or hypophysis, in the brain is an endocrine gland whose secretions control the other endocrine glands and systemically influence growth, metabolism, and hemodynamics^3^. This study on the pituitary gland sheds new light on resuscitation science to better explain the mechanisms of a post-CA syndrome.

The pituitary gland, consisting of the anterior (pars distalis) and posterior (pars nervosa) lobes, plays an important endocrinal role exerting significant influence over cardiovascular homeostasis^4^. The anterior pituitary gland is responsible for the production of hormones, including adrenocorticotropic hormone (ACTH), thyroid-stimulating hormone, growth hormone (GH), follicle-stimulating hormone, luteinizing hormone, and prolactin. These hormones modulate downstream effects through the hypothalamic-pituitary-target organ axis. The posterior lobe does not synthesize any hormones of its own; instead, it stores and secretes two hormones (vasopressin and oxytocin) produced by magnocellular neurosecretory neurons in the paraventricular nucleus of the hypothalamus and supraoptic nucleus^5^. Generally, the causes of pituitary insufficiency include pituitary or other intrasellar and parasellar tumors, surgical removal, infectious destruction, radiation-induced destruction, traumatic brain injury, subarachnoid hemorrhage, postpartum pituitary necrosis, and post-CA syndrome^6–8^.

Post-CA syndrome is considered to involve the hypothalamic–pituitary–adrenal (HPA) system as a part of systemic ischemia–reperfusion injury^8–10^. Compelling data from clinical and preclinical studies have revealed the association between post-CA adrenal insufficiency and increased risk of mortality and morbidity of CA patients^8,11^. However, none of these studies focused on the broad spectrum of the pituitary gland system, including vasopressin and other anterior pituitary gland hormones.

In this study, we investigated pituitary damage after CA. First, we evaluated copeptin (C-terminal portion of pro-arginine-vasopressin: CT-proAVP) as a pars nervosa hormone^12^, and GH and insulin-like growth factor-1(IGF-1) as pars distalis hormones^13^. In order to evaluate the time-dependent transportation of these hormones and their effects on the end-organs, we measured hormonal levels at several time points and in several samples including plasma and urine samples. We also assessed the correlation between the degree of pituitary damage and plasma makers to aim for a potential, non-invasive, indicator of pituitary damage. In addition, to evaluate the possibility of multimodal etiology of damaging process after CA, we examined an inflammatory indicator such as high mobility group box 1 (HMGB-1) and a renal function of electrolyte exchange and sodium-glucose cotransporter-2 (SGLT-2).

## Material and methods

Our research protocol and methods followed Guide for the Care and Use of Laboratory Animals and the Institutional Animal Care and Use Committees (IACUC) of the Feinstein Institutes for Medical Research approved the study protocol.

### Animal preparation

The details of the methods for a rat asphyxia CA model have been described previously^14^. In brief, male Sprague–Dawley rats (450–550 g, Charles River Laboratories) were anesthetized with 4% isoflurane (Isothesia, Butler-Schein AHS) and intubated with a 14-gauge plastic catheter (Surflo, Terumo Medical Corporation). The animals were mechanically ventilated (Ventilator Model 683, Harvard Apparatus, Holliston, MA, USA). A temperature probe was placed in the esophagus and the core temperature was maintained at 36.5 ± 1.0 °C. Neuromuscular blockade was achieved by slow intravenous administration of 2 mg/kg of vecuronium bromide (Hospira, Lake Forest, IL, USA). Asphyxia was induced in the rats by switching off the ventilator, and CA occurred 3 to 4 min after asphyxia started. CA remained completely untreated during 10 min of asphyxia. Mechanical ventilation was restarted at an FIO2 of 1.0, and manual cardiopulmonary resuscitation (CPR) was delivered to CA animals. Chest compressions were performed at a rate of 240 to 300 per minute. At 30 s after the beginning of CPR, a 20 μg/kg bolus of adrenaline was given. Mechanical ventilation was continued at an FIO2 of 1.0 up to 120 min after CA. Blood collection was obtained pre-CA (control), 30 and 120 min after CA. For tissue samples, rats were assigned into three groups: pre-CA (control) with surgical preparation; sacrificed at 30 min after CA; and 120 min after CA. Tissue samples were separately prepared for triphenyltetrazolium chloride staining (n = 8 for each group), immunohistochemistry (n = 6 for each group), and immunochemical assays (n = 8 for each group). The control group animals were given the surgical preparation described above except asphyxia and chest compression.

### Triphenyltetrazolium chloride staining of pituitary

Brain tissues were perfused transcardially through the left ventricle with a 4 °C extracellular immediately prior to the sampling. The harvested pituitaries were incubated for 30 min with 2% 2,3,5-triphenyltetrazolium chloride (TTC, Sigma Aldrich, St. Louis, MO, USA) at 37 °C and then fixed with 4% formalin in phosphate buffered saline (PBS)^15^. The stained pituitaries were photographed, and these were quantified using the BZ-800 Analyze software program (Keyence, Elmwood Park, NJ, USA). All values were expressed as the ratio of the lesion and the whole area^16^.

### Immunohistochemistry

Tissues were post-fixed overnight in a 4% formalin in PBS for three days at 4 °C and then cryoprotected overnight in a 30% sucrose in PBS at 4 °C. Subsequently, tissues were embedded in M-1 Embedding Matrix (Thermo Scientific, Kalamazoo, MI) and frozen in liquid nitrogen, cut serially (10 μm thickness) in a cryostat, and collected on glass slides. After washing with tris-buffered saline (TBS), the sections were immersed in 10% normal goat serum (Sigma-Aldrich Co., St. Louis, MO, USA) in TBS containing 1% bovine serum albumin (BSA, Sigma-Aldrich Co., St. Louis, MO, USA) for 2 h to block non-specific binding. For double immunostaining, the sections were incubated overnight with anti-growth hormone (GH) monoclonal antibody (Ab) (R&D Systems, Inc., Minneapolis, MN, USA) in combination with anti-copeptin (C-terminal portion of pro-arginine-vasopressin: CT-proAVP) polyclonal Ab (MyBioSource, Inc., San Diego, CA, USA) as the primary antibodies (Abs) at 4 °C. Alexa-555 labeled anti-mouse IgG (Invitrogen Co., Branford, CT, USA) and alexa-488 labeled anti-rabbit IgG (Invitrogen Co., Branford, CT, USA) were used as the secondary Abs. Sections were incubated with the secondary Ab at room temperature for 1 h and mounted using VECTORSHIELD Hard Set Mounting Medium with 4,6-Diamido-2-Phenylindole (DAPI, Vector Laboratories, Inc., Burlingame, CA, USA) as previously described^17^. Stained sections were observed under LSM 880 confocal imaging system (Carl Zeiss, Inc., Jena, Germany) and BZ-X800 all-in-one fluorescence microscope (Keyence, Elmwood Park, NJ, USA). We performed the analysis using the BZ-800 Analyze software program^18^.

### Immunochemical quantitative tests

The tissue homogenates were obtained as per our standard procedures. Protein contents were determined using a BCA protein kit (Pierce, Thermo Scientific, IL, USA) as per the manufacturer’s instruction. The numbers reported in this study have been expressed as relative to the protein content. Copeptin (G-biosciences, Saint Louis, MO, USA) of the pituitary, kidney, and plasma, IGF-1 (Thermo Scientific, Frederick, MD, USA) of the pituitary, kidney, and plasma, sodium-glucose cotransporter-2 (SGLT2, MyBioSource, San Diego, CA, USA) of the kidney, plasma, and urine, N-terminal portion of pro-brain natriuretic peptide (NT-proBNP, G-biosciences, Saint Louis, MO, USA) of the plasma, and high mobility group box-1 (HMGB-1, Shino-test, Sagamihara, Kanagawa, Japan) of the pituitary were measured with the enzyme-linked immunosorbent assay (ELISA) following the commercial protocols. The urinal sodium and glucose were measured by i-STAT (Abbott Laboratories, Abbott Park, IL, USA)^19^.

### Statistics

We reported data as mean and SD. For the comparison of the 3 groups, we used a 1-way analysis of variance (ANOVA) with post-hoc analysis using the Tukey test. Correlation coefficient (r) values were analyzed to measure the strength of the relationship between the multiple parameters. All statistical analyses were performed with JMP (version 10.1 software: SAS Institute, Cary, NC, USA). P-values less than 0.05 were considered significant.

## Results

### The expansion of damage and associated inflammatory process of the pituitary

**Figure 1:**
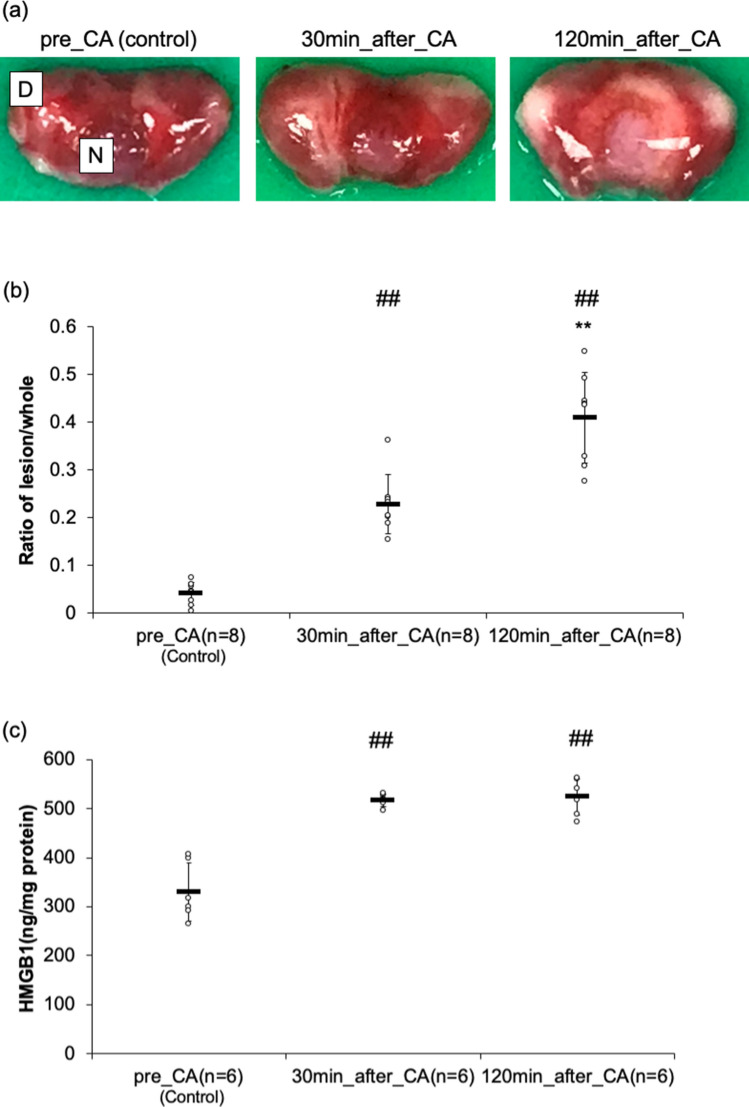
The evaluation of the pituitary damaged after asphyxial cardiac arrest (CA). (**a**) In order to evaluate the lesion volumes, the pituitaries were stained by 2% 2, 3, 5-triphenyltetrazolium chloride (TTC) at different time points. Every representative case from each group was shown. (**b**) We evaluated using the ratio of the lesion divided by the whole area. The results are indicated as mean ± SD. ^##^*p* < 0.01 compared with the pre-CA group. **p < 0.01 compared with 30 min post-CA group. (**c**) In order to determine the damaged molecular patterns in the pituitary, we evaluated the pituitary HMGB-1 level by enzyme-linked immunosorbent assay in CA rats. The results are shown as mean ± SD. ^##^*p* < 0.01 compared with the pre-CA group.

Asphyxia CA induced massive and gradual damage exhibited at TTC staining through 2 h of resuscitation. The damaged volumes were quantified and results have been summarized in Fig. 1. The ratio of lesion/whole volume significantly and gradually increased over time (pre-CA, 30 min post-CA, and 120minutes post-CA: 0.04 ± 0.02, 0.23 ± 0.06, and 0.41 ± 0.10 respectively). The damage gradually expanded from the pars distalis (D) into the pars nervosa (N) (Fig. 1).

Dynamic quantitative levels of HMGB-1 in the pituitary revealed that HMGB-1 immediately increased after resuscitation (pre-CA, 30 min post-CA and 120minutes post-CA 330 ± 59, 518 ± 13 and 524 ± 37 ng/mg protein respectively) (Fig. 1).

### The time course of CT-proAVP and GH levels in the pituitary

**Figure 2:**
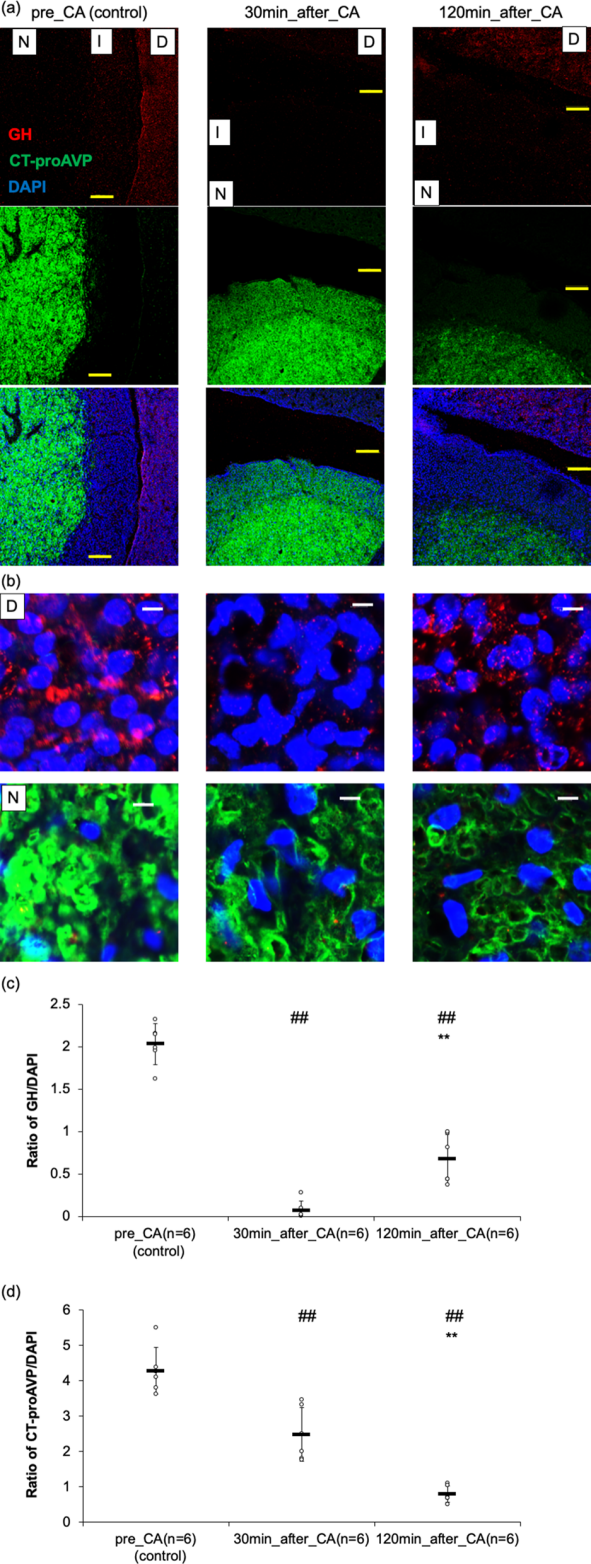
Time-dependent change of copeptin (C-terminal portion of pro-arginine-vasopressin: CT-proAVP) and growth hormone (GH) in the pars distalis (D), the pars nervosa (N), and the pars intermedia in pituitary of the asphyxial cardiac arrest (CA) rat. The pituitary sections were double-immunostained with anti-CT-proAVP and anti-GH antibodies, followed by AlexaFluor 555-labeled and AlexaFluor 488-labeled secondary antibodies respectively. 4, 6-Diamido-2-Phenylindole (DAPI) staining was performed to demonstrate the cell nuclei. LPF, low power field; HPF, high power field. (**a**) LPF of the pituitaries at different time points. Scale bars meant 100 um (yellow). (**b**) HPF of the pars D and the pars N in pituitaries at different time points. Scale bars meant 5um (white). (**c**) The ratio of GH divided by DAPI in pars N were measured using the BZ-800 Analyze software program. The results are indicated as mean ± SD. ^##^*p* < 0.01 compared with pre-CA group. **p < 0.01 compared with 30 min post-CA group. (**d**) The ratio of CTp-proAVP divided by DAPI in pars D were measured using the BZ-800 Analyze software program. The results are shown as mean ± SD. ^##^*p* < 0.01 compared with pre-CA group. **p < 0.01 compared with 30 min post-CA group.

Immunohistochemical studies of the pituitary revealed the presence of CT-proAVP decreased gradually and remarkably after resuscitation (Fig. 2). The evaluation of the ratio of CT-proAVP positive area divided by DAPI positive area counted in the pars nervosa (N) also revealed and confirmed the same trend significantly as above (pre-CA, 30 min post-CA, and 120 min post-CA: 4.28 ± 0.66, 2.49 ± 0706, and 0.80 ± 0.23, *p* < 0.01 respectively). In contrast, the presence of GH decreased immediately but recovered partially over time (Fig. 2). The evaluation of the ratio of GH positive area divided by DAPI positive area counted in the pars distalis (D) also indicated and confirmed the same trend significantly as above (pre-CA, 30 min post-CA, and 120 min post-CA: 2.03 ± 0.24, 0.07 ± 0.11, and 0.68 ± 0.29, *p* < 0.01 respectively).

### The time course of CT-proAVP and GH in the kidney

**Figure 3:**
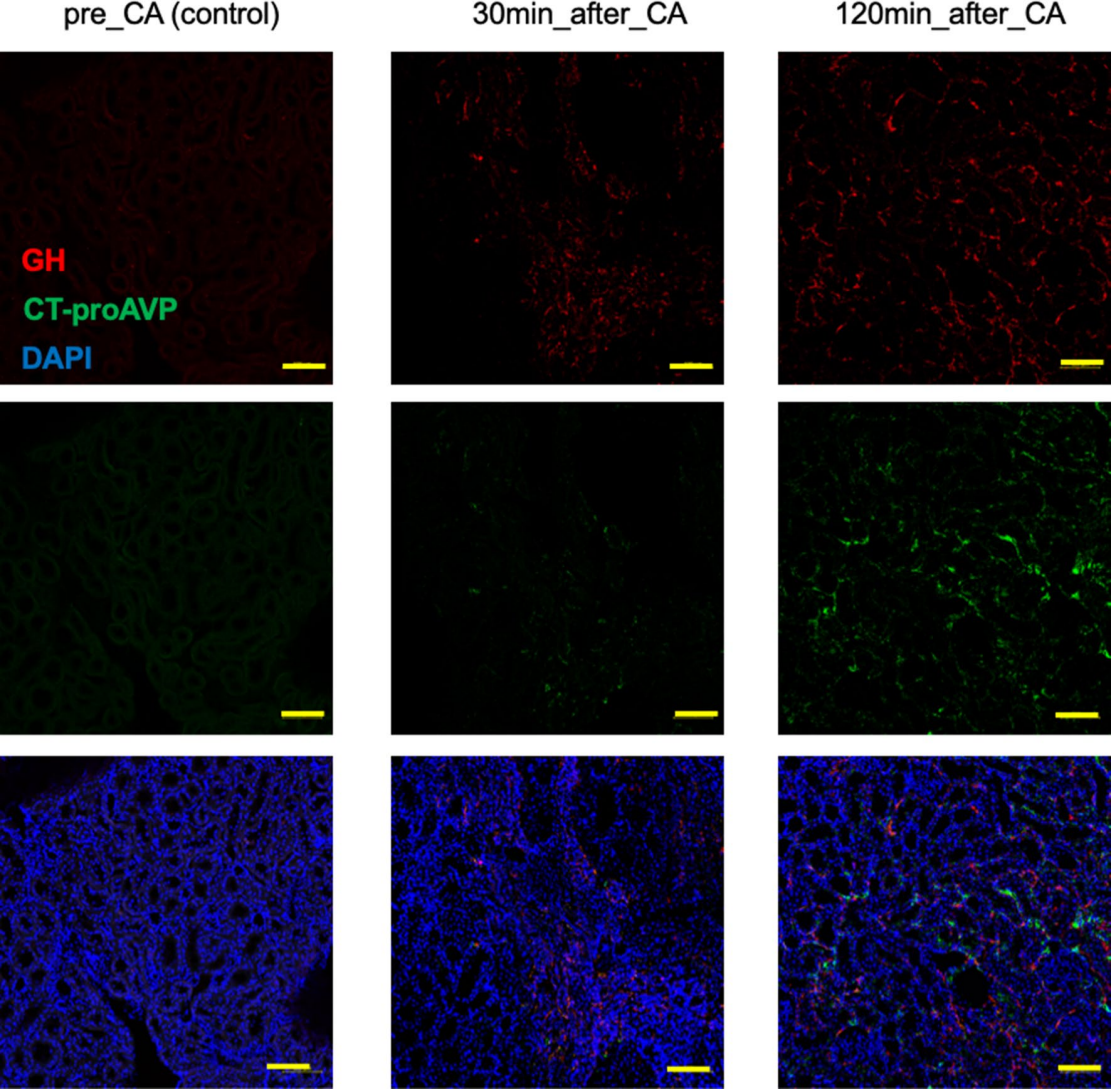
Time-dependent change of copeptin (C-terminal portion of pro-arginine-vasopressin: CT-proAVP) and growth hormone (GH) in the kidney of the rat with asphyxial cardiac arrest. The kidney sections were double-immunostained with anti-CT-proAVP and anti-GH antibodies, followed by AlexaFluor 555-labeled and AlexaFluor 488-labeled secondary antibodies respectively. 4, 6-Diamido-2-Phenylindole (DAPI) staining was performed to show the cell nuclei. LPF, low power field. LPF of the pituitaries at different time points. Scale bars meant 100 um (yellow). There seemed a difference between the absorbed site of CT-proAVP and that of GH.

Immunohistochemical studies of the kidney revealed the absence of CT-proAVP and GH at pre-CA and gradual increase after resuscitation (Fig. 3). A different uptake pattern between CT-proAVP and GH was noted. Increased GH was observed from 30 min after CA, while that of CT-proAVP occurred 120 min after CA. GH uptake in the kidney increased earlier than CT-proAVP after resuscitation (Fig. 3).

### Dynamic quantitative levels of CT-proAVP and IGF-1 by ELISA

**Figure 4:**
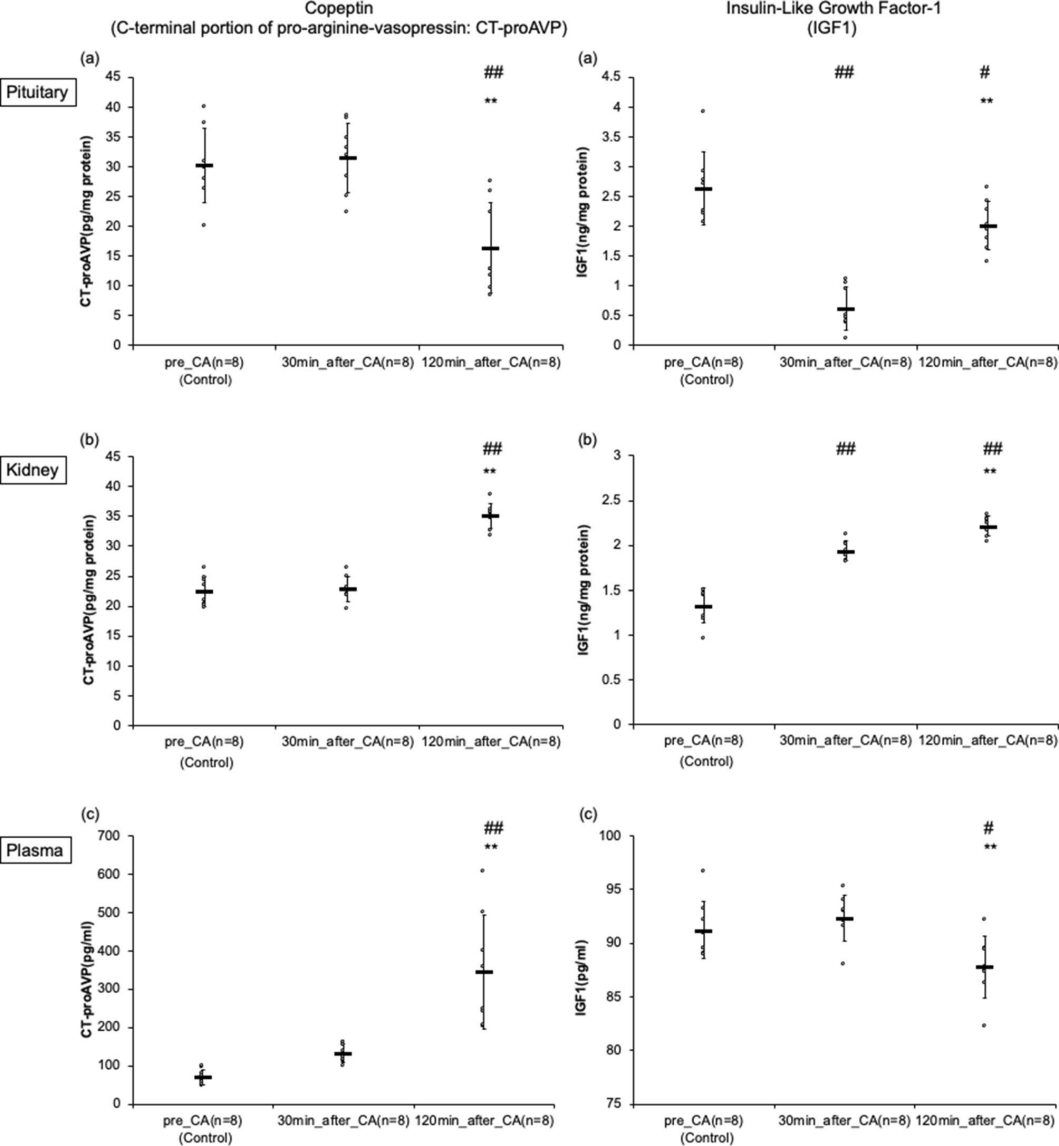
Dynamic quantitative measures of copeptin (C-terminal portion of pro-arginine-vasopressin: CT-proAVP) of the pituitary, kidney, and plasma, insulin-like growth factor-1 (IGF1) of the pituitary, kidney, and plasma by enzyme-linked immunosorbent assay in asphyxial cardiac arrest (CA) rats. (**a**) This indicates the results of the pituitary. ^##^*p* < 0.01 compared with the pre-CA group. **p < 0.01 compared with 30 min post-CA group. (**b**) This shows the results of kidney. ^##^*p* < 0.01 compared with the pre-CA group. **p < 0.01 compared with 30 min post-CA group. (**c**) This shows the results of the plasma. ^##^*p* < 0.01 compared with the pre-CA group. **p < 0.01 compared with 30 min post-CA group. Numbers are expressed as mean ± SD.

In the pituitary, CT-proAVP levels gradually decreased (pre-CA, 30 min post-CA, and 120 min post-CA: 30.2 ± 6.2, 31.5 ± 5.9, and 16.3 ± 7.6 pg/mg protein, *p* < 0.01 respectively). Conversely, IGF-1 levels immediately decreased but partially recovered (pre-CA, 30 min post-CA, and 120 min post-CA: 2.63 ± 0.61, 0.62 ± 0.36, and 2.01 ± 0.41 ng/mg protein, *p* < 0.01 respectively). This trend was similar to that of the immunohistochemistry studies of the pituitary (Fig. 4).

In the kidney, CT-proAVP levels increased at 120 min after CA (pre-CA, 30 min post-CA, and 120 min post-CA: 22.5 ± 2.5, 22.9 ± 2.1, and 35.1 ± 2.2 pg/mg protein, *p* < 0.01 respectively). Conversely, IGF-1 levels increased soon after resuscitation and remained high over time (pre-CA, 30 min post-CA, and 120 min post-CA: 1.33 ± 0.19, 1.94 ± 0.11, and 2.22 ± 0.11 ng/mg protein, *p* < 0.01 respectively). This trend was similar to that of the immunohistochemistry studies of the kidney (Fig. 4).

In the plasma samples, CT-proAVP levels gradually increased (pre-CA, 30 min post-CA, and 120 min post-CA: 70.1 ± 19.9, 132 ± 24, and 344 ± 149 pg/ml, *p* < 0.01 respectively). Conversely, IGF-1 levels decreased at 120 min after CA (pre-CA, 30 min post-CA, and 120 min post-CA: 91.2 ± 2.7, 92.4 ± 2.1, and 87.8 ± 2.9 pg/ml, *p* < 0.01 respectively) (Fig. 4).

### Dynamic quantitative levels of SGLT2 and NT-proBNP by ELISA

**Figure 5:**
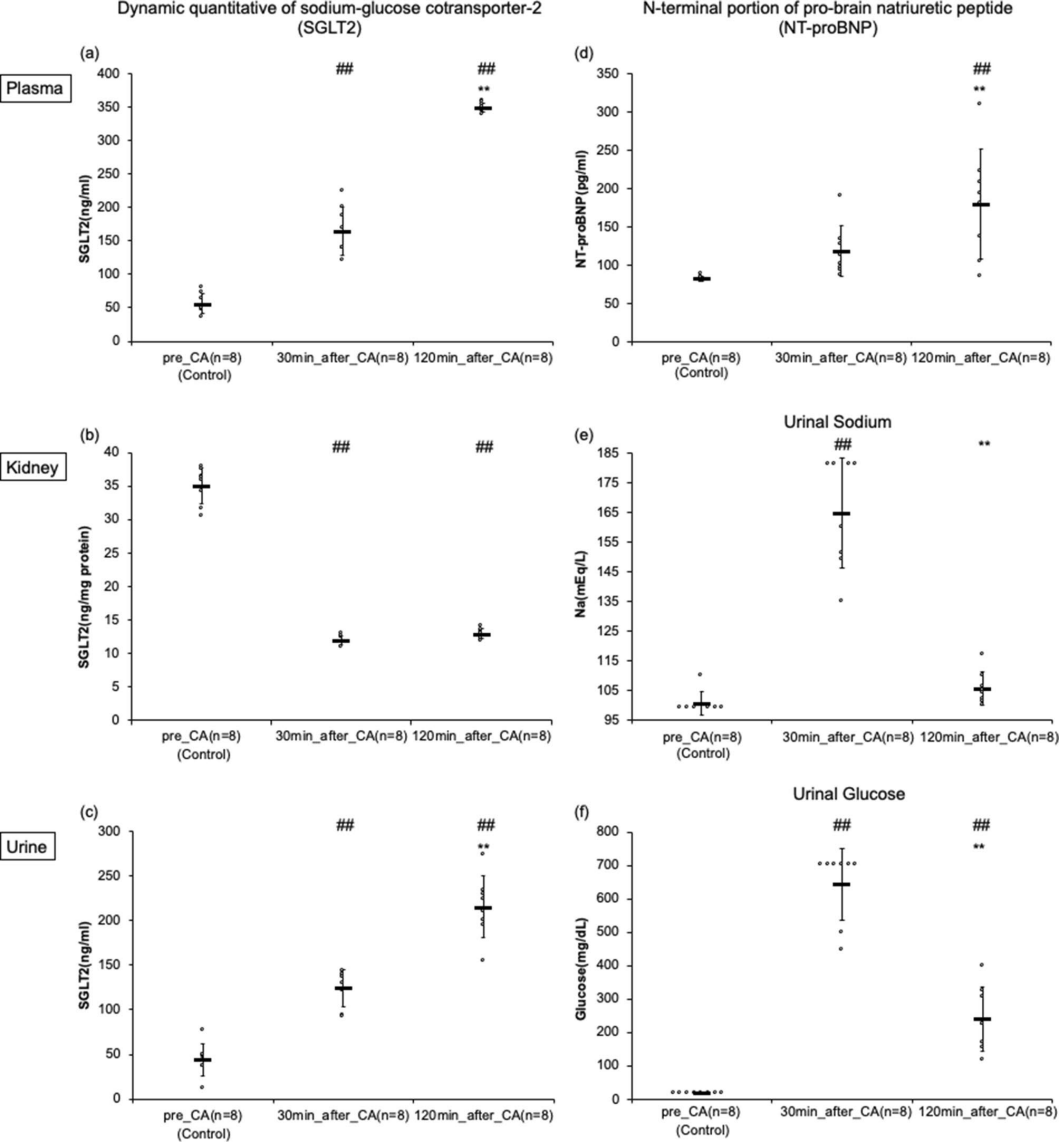
(**a**)–(**d**) Dynamic quantitative measures of sodium-glucose cotransporter-2 (SGLT2) of the kidney, plasma, and urine, N-terminal portion of pro-brain natriuretic peptide (NT-proBNP) of plasma by enzyme-linked immunosorbent assay in asphyxial cardiac arrest (CA) rats. (**a**) This shows the results of the plasma. ^##^*p* < 0.01 compared with the pre-CA group. **p < 0.01 compared with 30 min post-CA group. (**b**) This demonstrates the results of the kidney. ^##^*p* < 0.01 compared with the pre-CA group. **p < 0.01 compared with 30 min post-CA group. (**c**) This indicates the urine results. ^##^*p* < 0.01 compared with the pre-CA group. **p < 0.01 compared with 30 min post-CA group. (**d**) This shows the results of plasma as mean ± SD. ^##^*p* < 0.01 compared with the pre-CA group. **p < 0.01 compared with 30 min post-CA group. (**e**)–(**f**) Dynamic quantitative measures of sodium and glucose levels in the urine. (**e**) This shows the result of urine sodium. ^##^*p* < 0.01 compared with the pre-CA group. **p < 0.01 compared with 30 min post-CA group. (**f**) This shows the result of urine glucose. ^##^*p* < 0.01 compared with the pre-CA group. **p < 0.01 compared with 30 min post-CA group. Numbers are expressed as mean ± SD.

In the plasma samples, SGLT2 levels gradually increased (pre-CA, 30 min post-CA, and 120 min post-CA: 56.2 ± 14.5, 165 ± 36, and 349 ± 7.0 ng/ml, *p* < 0.01 respectively). NT-proBNP also gradually increased (pre-CA, 30minutespost-CA, and 120 min post-CA: 81.9 ± 2.9, 118 ± 33, and 180 ± 72 pg/ml, *p* < 0.01 respectively) (Fig. 5).

In the kidney, SGLT2 levels decreased immediately after resuscitation (pre-CA, 30 min post-CA, and 120 min post-CA: 35.0 ± 2.7, 11.9 ± 0.6, and 12.9 ± 0.7 ng/mg protein, *p* < 0.01 respectively) (Fig. 5).

In the urine samples, SGLT2 gradually increased (pre-CA, 30 min post-CA, and 120 min post-CA: 5.51 ± 2.22, 15.6 ± 2.6, and 26.9 ± 4.3 ng/ml, *p* < 0.01 respectively) (Fig. 5).

### Dynamic quantitative urine levels of sodium and glucose

Urine sodium levels immediately increased after resuscitation and dropped thereafter (pre-CA, 30 min post-CA, and 120 min post-CA: 101 ± 4, 165 ± 19, and 106 ± 6 mmol/L, *p* < 0.01 respectively) (Fig. 5). Urine glucose levels also immediately increased after resuscitation and dropped thereafter (pre-CA, 30 min post-CA, and 120 min post-CA: 19 ± 0, 644 ± 107, and 240 ± 96 mg/dL, *p* < 0.01 respectively) (Fig. 5).

### Dynamic quantitative plasma levels of Cortisol and Adrenocorticotropic hormones

**Figure 6:**
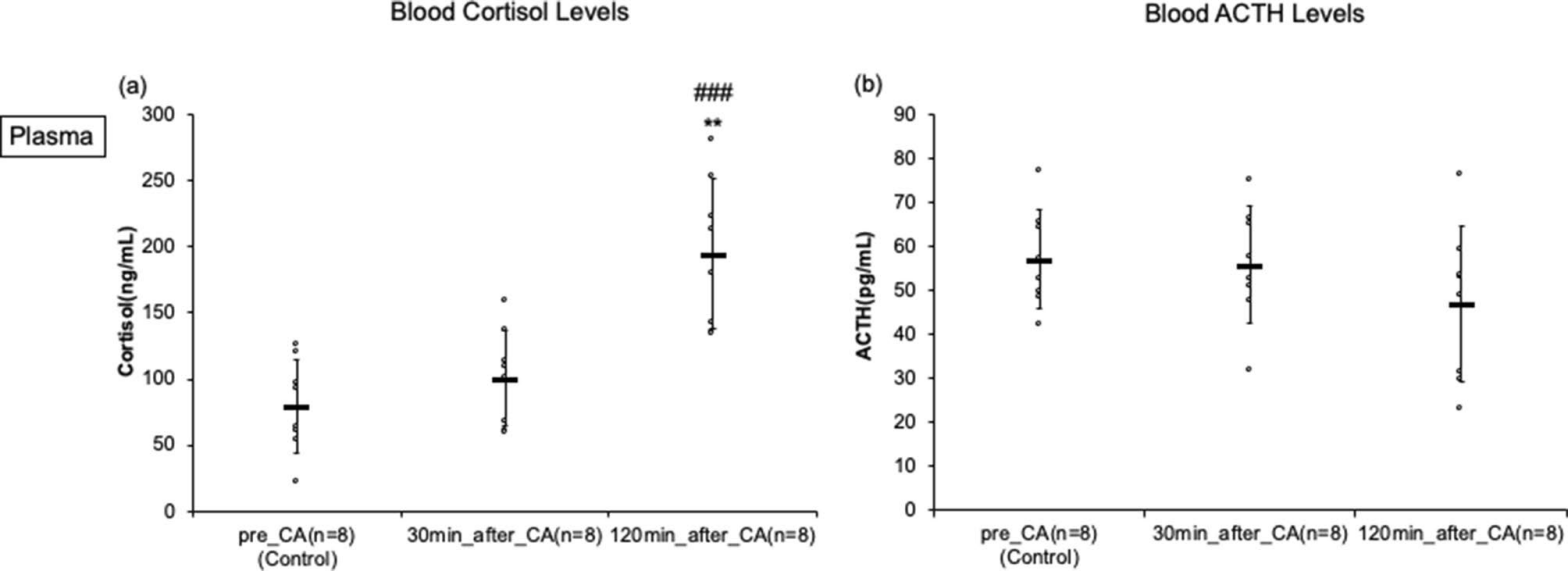
Cortisol and ACTH plasma levels by enzyme-linked immunosorbent assay in asphyxial cardiac arrest (CA) rats. (**a**) blood cortisol level. (**b**) blood ACTH level. ^###^*p* < 0.001 compared with the pre-CA group. **p < 0.01 compared with 30 min post-CA group.

Plasma cortisol levels gradually increased and demonstrated a significant increase at 120 min after resuscitation (pre-CA, 30 min post-CA, and 120 min post-CA: 79 ± 35, 101 ± 36, and 195 ± 56 ng/mL, *p* < 0.001) (Fig. 6). There were statistically no differences in ACTH levels (pre-CA, 30 min post-CA, and 120 min post-CA: 57 ± 11, 56 ± 14, and 47 ± 18 pg/mL) (Fig. 6).

### Evaluation of the correlation between pituitary damage and plasma markers

We evaluated the correlation between the degree of pituitary damage and the level of plasma biomarkers at 120 min after resuscitation. CT-proAVP revealed a significant positive correlation with pituitary lesion ratio of TTC (*r* = 0.771,* p* = 0.025). IGF-1 also demonstrated a significant negative correlation with pituitary lesion ratio (*r* = −0.775, *p* = 0.024), while NT-proBNP and SGLT2 did not indicate any significance (*r* = −0.225, *p* = 0.592; *r* = −0.021, *p* = 0.9611 respectively) (Supplemental Digital Content 1).

## Discussion

This is the first study indicating the macroscopic/pathological damage of the pituitary gland following CA and resuscitation. We investigated the broad spectrum of hormonal dysfunction post-CA. In this study, TTC staining revealed the extent of the damage in the pituitary gland after resuscitation and the dynamic of its expansion from pars distalis into the pars nervosa over the initial 2 h of resuscitation^20^. Our macro findings with the TTC staining showed a good correlation with an increase of HMGB-1, which is a well-known marker of inflammatory damaged molecular patterns^17^.

Vasopressin is a hormone, which terminates in the pars nervosa, with multiple functions including endocrine, hemodynamic, and osmoregulatory effects. Endogenous vasopressin levels in patients undergoing CPR were reported significantly higher in those who survived than those who did not regain spontaneous circulation^21^. Other observations revealed that vasopressin levels increase in different states such as shock, heart failure, and sepsis^22,23^. However, vasopressin is known as being unstable in isolated plasma even when stored at − 20 °C, and more than 90% of AVP in the circulation is bound to platelets. Thus, these limitations preclude its routine use in clinical settings^24^.

Copeptin (CT-proAVP) is a 39-aminoacid glycopeptide^25^. Vasopressin and CT-proAVP share an extremely good correlation in plasma samples of healthy individuals and patients, and CT-proAVP is biologically more stable than AVP. Thus, CT-proAVP is used as a surrogate marker for vasopressin^26^. Compelling data indicated copeptin as an excellent predictor of outcomes of patients with heart failure and acute myocardial infarction (AMI); its accuracy was even superior to that of NT-proBNP^27,28^. Others also indicated that copeptin possessed the potential to be a surrogate marker for acute lung injury, glucose abnormalities with AMI, birth asphyxia, multiple organ dysfunction, death in the intensive care units, cardiac stress after generalized convulsive seizures, and neurological dysfunction and mortality in post-CA syndrome^29–33^. Moreover, CT-proAVP was reported to be associated with hypopituitarism^34^. Our results are in line with the findings of other researchers. We found that the plasma CT-proAVP level had the best correlation with the pituitary damage when measured at 120 min after resuscitation. Plasma biomarker measurement is non-invasive, and, moreover, it allows clinicians to estimate the pituitary damage and possibly indicate the ongoing pathophysiology, such as post-CA hypopituitarism.

Our immunohistochemistry data displayed that reductions of CT-proAVP and GH in the pituitary gland correspond to our macro findings in light of the damage expansion pattern that occurred over time. Additionally, CT-proAVP and IGF-1 levels in the plasma and kidney suggested that these hormones were released from the pituitary gland and transported to the kidney via the blood after resuscitation. We observed a phenotypic validity of secretion patterns between CT-proAVP and GH. CT-proAVP gradually decreased from the pars nervosa over 2 h of resuscitation and was continuously transported to the kidneys via the blood, whereas GH was rapidly decreased from the pars distalis and transported, and then slightly recovered in the pituitary, resulting in a gradual increase in the kidney over time. Immunohistochemistry studies of the kidney also supported the consistency of our findings.

Stress-induced HPA-axis activity and concomitant inflammatory responses are of great importance in resuscitation science, and there is abundant clinical evidence supporting its significance in the pathophysiology of post-CA syndrome^35–39^. ACTH (pars distalis hormone) and cortisol levels were measured in the plasma samples. There were no significant differences or alteration patterns observed in the ACTH, while the cortisol levels increased at 120 min after resuscitation. Given our finding of the increased HMGB-1, the elevation of cortisol levels may be inferred as the evidence of activated inflammatory pathways, which may or may not be linked to the ACTH level in early post-CA period. The consistent data showed the increase in cortisol levels after CA from the human studies, but no study has tested the dynamics of these hormones in rats. Zhao et al. used a murine CA model and reported the results of ACTH and corticosterone levels, demonstrating the overexpression of HPA with concomitant inflammatory responses observed 1–3 days after CA^40^. In our CA rat model, pars distalis showed an early recovery pattern—within 2 h after CA—and therefore, it is plausible that the anterior gland system, including HPA, can be overexpressed in the late period of post-CA responses. However, due to the paucity of evidence, it is difficult to arrive at any conclusions, and further studies on long-term pituitary damage are necessary.

It is crucial to understand the blood supply of the pituitary gland, which might be the key to explaining the phenotypic validity of the damaging process after resuscitation. The pars nervosa is supplied by the inferior hypophysea. The pars distalis is supplied by the superior hypophyseal and is also supplied by portal vessels that originate dorsally at capillaries from the median eminence and travel along the stalk. Around 70% to 90% of the pars distalis supply comes from major portal vessels, in addition to the arterial supply, resulting in the rich vascular system^41^. It is inferred that the difference in the vascular system and the amount of blood supply may explain the phenotypic validity of the damaging process after resuscitation. It is also critical to understand other possible factors that can affect the cerebral blood flow in CA. For example, other phenotypes of CA, e.g., ventricular fibrillation may show a different pattern or time-course of pituitary damaging process as the cerebral blood flow is different^42^ and the regional (local) blood flow in the brain is plausibly varied based on the etiology and can affect the outcomes of CA^43^.

In order to evaluate the kidney function affected by the dynamic change in vasopressin and GH levels, we next examined urine glucose and sodium levels. These levels increased immediately after resuscitation but soon recovered to the baseline. Vasopressin generally induces the reabsorption of sodium, promotes renal water reabsorption, and concentrates urine. However, our findings from the urine samples were not completely consistent with this general understanding. Because CA is a systemic level ischemia/reperfusion injury, it is plausible that the kidney responds to the elevated vasopressin/GH unusually. In an attempt to understand the paradox, we then examined sodium-glucose cotransporters (SGLTs). SGLTs are important mediators of glucose uptake across apical cell membranes. The proximal tubule cell uses ATP molecules to replace sodium and potassium irons across the basolateral membrane, and then, the SGLT protein transports glucose across the apical membrane. Therefore, sodium and glucose are moved in the same direction across the membrane^44^. Our results revealed that SGLT2 level in the kidney decreased after resuscitation, while SGLT2 levels in the plasma and urine gradually increased over time. Therefore, it can be inferred that SGLT2 is gradually expressed after resuscitation and affects glucose/sodium reabsorption interacting vasopressor in the kidney as a compensatory response to the ischemia/reperfusion injuries.

This study has several limitations. First, further individual examinations of pars distalis and pars nervosa are needed to investigate the area-specific damage in the pituitary gland. Second, we only studied post-arrest pituitary damage in rats, and the novel findings reported in this article may not be applicable to humans because of the difficulties in translating findings from rodents to humans^45^. Moreover, other influences of hormonal or protein dynamics can exist, and species-dependent responses may cause variations in the interactions among hormones/proteins. For instance, Ito et al. conducted a clinical observational study and demonstrated a correlation between blood ACTH and vasopressin levels in CA patients^37^, which our model did not find. It can be inferred that the difference between species or the timing of the blood samples led to this; however, due to the paucity of existing evidence on ACTH, vasopressin, and other pituitary hormones from human or animal studies of CA, this has still not been clearly elucidated. More evidence is thus required. Testing larger gyrencephalic species, such as swine and non-human primates, is also critical. Third, the influence of the CPR model cannot be completely excluded. To the best of our knowledge, no studies have explored the effect of chest compression on pituitary function and its interactions with upstream/downstream hormonal systems. However, there are other acute injury models, such as subarachnoid hemorrhage^46^ and stroke^47^, that used rats and showed an activation of the HPA axis. Therefore, it can be inferred that any alteration of the pituitary function may be identified as a result of the stress response, which includes CA, chest compression, and subsequent post-CA responses. Finally, we could not determine whether the pituitary damage was reversible or irreversible, as in the penumbra in the cortex. Therefore, future research should focus on long-term pathophysiological changes and hormonal dysfunction.

## Conclusions

We found that CA followed by resuscitation damaged the pituitary gland. Phenotypic validity was obtained of damaging patterns between the pars distalis and pars nervosa over the initial 120 min of resuscitation. The level of CT-proAVP at 120 min after CA was highly associated with the degree of pituitary damage. Therefore, CT-proAVP may have the potential for clinical use in monitoring pituitary damage after cardiac arrest.

## Supplementary Information

Supplementary Table 1.

## Data Availability

The data supporting this study would be made available by the corresponding author upon reasonable request.
