# Supplementary material for: The evaluation of pituitary damage associated with cardiac arrest: An experimental rodent model
Source: Sci Rep. 2021 Jan 12;11:629. doi: 10.1038/s41598-020-79780-3 (PMC7804952; doi:10.1038/s41598-020-79780-3)
Supplement: Supplementary file 1 — Supplementary Table 1. [file 41598_2020_79780_MOESM1_ESM.docx]

**Supplemental Materials**

The evaluation of pituitary damage associated with cardiac arrest- an experimental rodent model

Authors: Yu Okuma^1^, MD, PhD; Tomoaki Aoki, MD, PhD^1^; Santiago Miyara, MD^1^; Kei Hayashida^1^, MD, PhD; Mitsuaki Nishikimi^1^, MD; Ryosuke Takegawa^1^, MD, PhD; Tai Yin^1^, MD, PhD; Junhwan Kim^1^, PhD; Lance B. Becker^1,2^, MD, FAHA; and Koichiro Shinozaki^1,2^, MD, PhD;

^1^ The Feinstein Institutes for Medical Research, Northwell Health, Manhasset, NY, USA

^2^ Department of Emergency Medicine, North Shore University Hospital/ Long Island Jewish Medical Center, Northwell Health, Manhasset, NY, USA

Corresponding to:

Koichiro Shinozaki

The Feinstein Institutes for Medical Research, Northwell Health

350 Community Dr., Manhasset, NY, 11030

Telephone: 516-562-0309

E-mail: [shino@gk9.so-net.ne.jp](mailto:shino@gk9.so-net.ne.jp)

**Supplemental Digital Content 1. Relationship between pituitary damage and plasma markers.**

|  |  | Correlation | Lower 95% | Upper 95% | P value |
| --- | --- | --- | --- | --- | --- |
| CT-proAVP | TTC | 0.7709 | 0.145 | 0.9562 | 0.0251 |
| IGF1 | TTC | -0.7753 | -0.9571 | -0.1556 | 0.0238 |
| SGLT2 | TTC | -0.0208 | -0.715 | 0.6941 | 0.9611 |
| NT-proBNP | TTC | -0.2251 | -0.8025 | 0.57 | 0.592 |
